# Highly purified chondroitin sulfate: a literature review on clinical efficacy and pharmacoeconomic aspects in osteoarthritis treatment

**DOI:** 10.1007/s40520-020-01643-8

**Published:** 2020-07-07

**Authors:** Jean-Yves Reginster, Nicola Veronese

**Affiliations:** 1 Division of Public Health, Epidemiology and Health Economics, University of Liège, Liège, Belgium; 2 WHO Collaborating Center for Public Health Aspects of Musculoskeletal Health and Aging, University of Liège, Liège, Belgium; 3 Chair for Biomarkers of Chronic Diseases, Biochemistry Department, College of Science, King Saud University, Riyadh, Kingdom of Saudi Arabia; 4 Geriatric Unit, Department of Internal Medicine and Geriatrics, University of Palermo, 90100 Palermo, Italy

**Keywords:** Chondroitin sulfate, Osteoarthritis, Knee osteoarthritis, Hip osteoarthritis, Hand osteoarthritis, Economic analysis

## Abstract

Osteoarthritis (OA) is the most prevalent musculoskeletal disease and a major cause of negative relevant outcomes, associated with an ever-increasing societal burden. Pharmaceutical-grade chondroitin sulfate (CS) was repeatedly reported to reduce pain and improve function in patients with OA. This article aims to review the evidence for the role of highly purified (hp) CS (Condrosulf^®^, IBSA) in the treatment of OA. We collected and reported evidence concerning (1) efficacy of hpCS 800 mg/day in the treatment of OA affecting the knee, hand and hip; (2) efficacy and safety of hpCS 1200 mg/day also in the oral gel formulation; (3) the safety profile of hpCS; (4) the difference of hpCS and pharmaceutical-grade formulations versus food supplements; (5) pharmacoeconomic added value of hpCS. The data support that hpCS is an effective and safe treatment of OA, with its effect already evident at 30 days; in addition, its beneficial action is prolonged, being maintained for at least 3 months after the drug is discontinued. Full safety reports’ analyses confirm that CS is safe to use and has almost no side effects, in particular, it showed better gastrointestinal tolerance if compared with non-steroidal anti-inflammatory drugs (NSAIDs). Moreover, the therapeutic strategy has proved to be cost-effective: treatment with CS reduced the use of NSAIDs and their side effects.

## Introduction

**Fig. 1:**
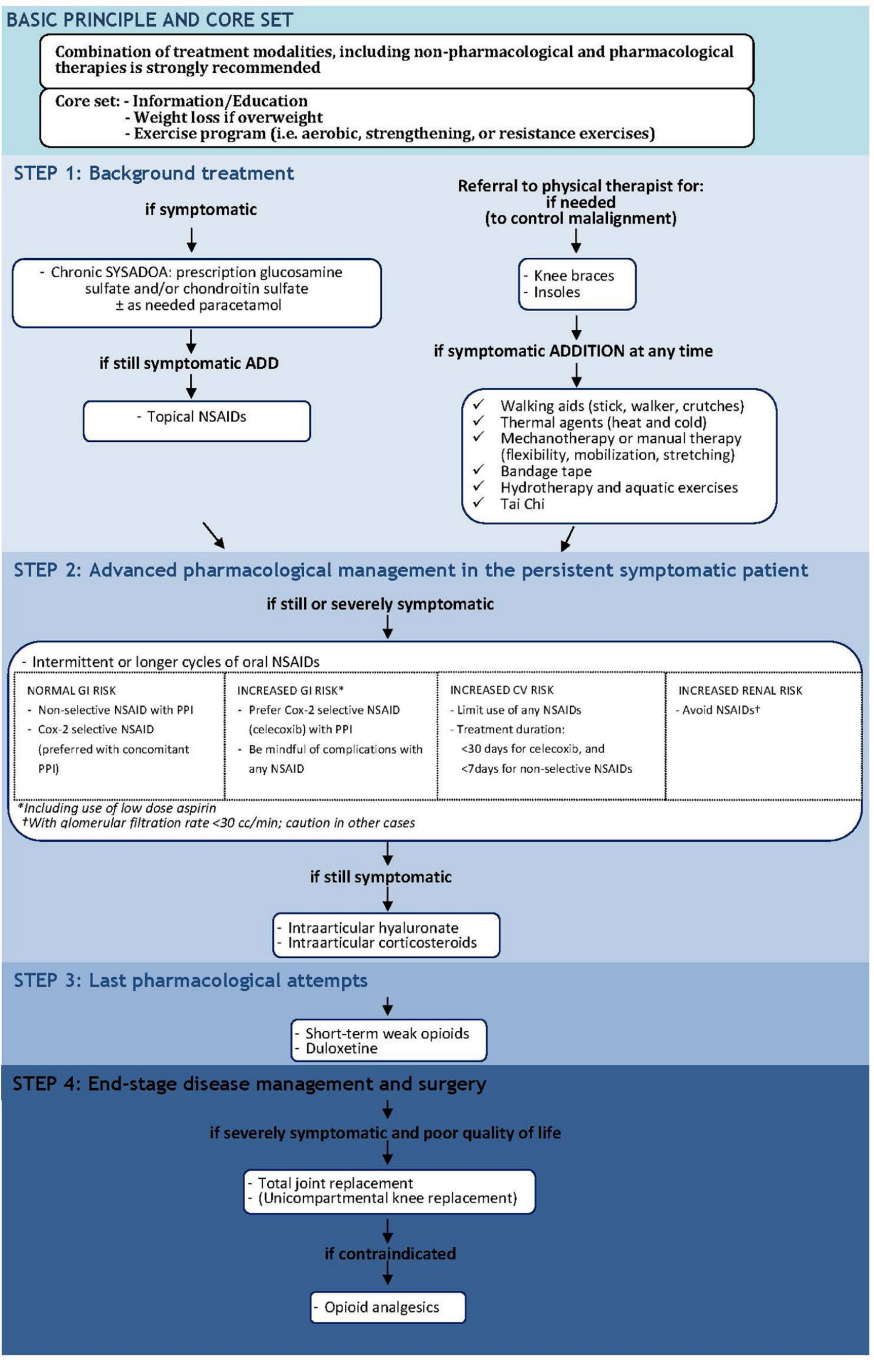
Updated ESCEO stepwise treatment algorithm for knee osteoarthritis. COX-2, cyclooxygenase-2; CS, chondroitin sulfate; CV, cardiovascular; GI, gastrointestinal; GS, glucosamine sulfate; IA, intra-articular; NSAID, non-steroidal anti-inflammatory drug; PPI, proton pump inhibitor; SYSADOA, symptomatic slow-acting drugs in osteoarthritis; OA, osteoarthritis. Reproduced from Bruyère et al. [10] with permission from BMJ Publishing Group Ltd

Osteoarthritis (OA) is a chronic inflammatory degenerative arthropathy that most commonly affects the joints in the knees, hands, feet, and spine; it is also relatively common in other joints such as the shoulder and hip joints and potentially affects all synovial joints [1, 2]. OA is the most common form of arthritis and is a leading cause of morbidity and chronic disability. Prevalence of OA increases with age: worldwide estimates are that 9.6% of men and 18.0% of women over 60 years have symptomatic OA. Eighty percent of people affected by OA experience limitations in movement, and 25% cannot perform their major daily life activities [3]. The main pathophysiological event is the thinning of cartilage in joints which results in bones rubbing together, causing stiffness, pain, and impaired movement with reduced quality of life and significant social and economic burden [4, 5]. Although research has focused primarily on the alterations in joint cartilage and synovial film, there is increasing evidence of involvement of all joint tissues [6]. The main goals in OA treatments are to relief pain, to slow the progression of joint structure modifications and to improve functional limitation and quality of life [7, 8]. Global strategies aim to reduce the burden of musculoskeletal disease and promote healthy ageing tailored to meet the individual patient’s needs [9]. The recent European Society for Clinical and Economic Aspects of Osteoporosis, Osteoarthritis and Musculoskeletal Diseases (ESCEO) guideline for the management of OA [10] recommend a combination of non-pharmacologic and pharmacologic measures (Fig. 1).

**Fig. 2:**
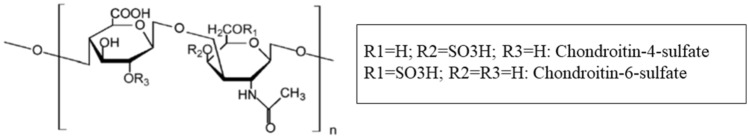
Formula of CS monomer

Fast-acting symptom modifying drugs include traditional analgesics (e.g*.* paracetamol, tramadol etc.), non-steroidal anti-inflammatory drugs (NSAIDs) and intra-articular corticosteroids. These substances induce a fast relief of symptoms and improve joint function [11]. However, they are not devoid of toxicity and when used chronically their gastrointestinal, liver, kidney and haematological side effects (sometimes serious) can be clinically important and associated to high costs, particularly in the elderly. Slow-acting drugs for the treatment of OA are defined as symptom modifying (Symptomatic Slow Acting Drugs of OA, SYSADOA). This group includes drugs that can be administered systemically (e.g*.* chondroitin sulfate CS, glucosamine GlcN sulfate, diacerein), as well as substances that can be administered intra-articularly (hyaluronic acid). Chondroitin sulfate oral supplementation is recommended by ESCEO and other European guidelines [10, 12, 13] as background treatment to reduce joint pain and improve functional impairment in OA patients. Chemically, CS monomer is a disaccharide molecule constituted by *N*-acetylgalactosamine and glucuronic acid; The sulfate group in CS can be linked to the galactosamine moiety in two positions—4 or 6—which explains the existence of two isomers (Fig. 2).

Chondroitin sulfate is usually derived from bovine, porcine, chicken or fish cartilage sources by extraction and purification procedures. Several clinical studies, as reported in the review by Chevalier and Conrozier [11] the meta-analysis by Hochberg et al. [15], Hochberg [15] and Honvo et al. [16, 17] demonstrated that CS at the marketed unit dose strengths (400 mg, 800 mg and 1200 mg) exerts an important activity on the mitigation of pain caused by OA, particularly knee, hand and hip OA; CS induces an improvement of algo-functional scores with a very low risk of toxicity reported in post-marketing surveillance data.

Considering the important role played by CS in the treatment of OA, we aimed to review the existing evidence on highly purified chondroitin sulfate (hpCS) (Condrosulf^®^ IBSA), a prescription drug containing highly purified chondroitins 4 and 6 sulfate in a concentration not less than 95%. We grouped the evidence by involved joint, adding specific discussion for the new dosage of hpCS 1200 mg, for the comparison with food supplements and for pharmacoeconomic aspects.

## Materials and methods

The MEDLINE and PubMed databases were searched for randomized controlled trials (RCTs), meta-analyses, systematic reviews and review articles on hpCS in OA published between 1992 and 2020. The search strategy was based on carefully constructed review questions and was performed using the appropriate clinical terms to identify all papers containing information on hpCS efficacy and safety and to address specific questions relevant to the dosage as well as pharmacoeconomic aspects. PICO evidence-based model [18] was used for framing a question, locating, assessing, evaluating and repeating as needed. PICO keywords are focused on:

- Problem/patient/population: people with osteoarthritis.
- Intervention: chondroitin sulfate.
- Comparison: placebo/no intervention.
- Outcomes: pain and function.

The literature searches included the terms: ‘chondroitin sulfate’, ‘pharmaceutical-grade’, ‘highly purified’, ‘osteoarthritis’ ‘knee osteoarthritis’, ‘hip osteoarthritis’, ‘hand osteoarthritis’, ‘food supplement’ ‘pharmacoeconomy’. The references of retrieved paper were manually searched for additional relevant articles and guidelines and OA treatment recommendations were also considered.

### Pharmacokinetics

The structure and characteristics of hpCS, such as molecular mass, charge density (in terms of electrostatic properties related to sulfated and nonsulfated disaccharides) and cluster of disulfated disaccharides can strongly influence its absorption and bioavailability. The pharmacokinetic (PK) characteristics of oral hpCS were evaluated in healthy volunteers [19, 20]. In a study by Conte et al. [20] hpCS was given to 12 healthy volunteers in fasting conditions as a single dose or two 400 mg doses at a 12-h interval. Table 1 summarizes the results of the main PK parameters in this study.

**Table 1:** Pharmacokinetic parameters after oral administration of 800 mg hpCS. Modified from Conte et al. [20]

PK parameter	800 mg single dose (mean ± SD)	400 mg + 400 mg dose (mean ± SD)
*C*_max_ (μg/ml)	2.6 ± 0.5	1.2 ± 0.2^a^
*t*_max_ (h)	5.0 ± 1.0	5.2 ± 1.0^a^
*t*½ (h)	10.3 ± 6.8	10.3 ± 2.5^b^
AUC_0–12h_ (μg h/ml)	23.9 ± 4.2	10.6 ± 1.7
AUC_∞_ (μg h/ml)	46.8 ± 10.1	37.3 ± 9.4

^a^After administration of the first 400 mg dose

^b^After administration of the second 400 mg dose

Twenty-four hours after oral administration, a high concentration was found in the intestine, liver and kidneys, organs involved in the breakdown and the excretion of oligo- and polysaccharides, but also in the synovial fluid and cartilages, where the molecule tends to accumulate [21, 22].

### Pharmacodynamics

The pharmacodynamics of CS is extensively studied. Bassleer et al. [23] found that in chondrocytes, hpCS antagonizes interleukin-1(IL-1)-induced increases in p38 mitogen-activated protein kinase (p38MAPK) and signal-regulated kinase 1/2 (Erk1/2) phosphorylation and decreases in nuclear factor-B (NFB) nuclear translocation and as a consequence reduced formation of pro-inflammatory cytokines, IL-1 and TNF, and pro-inflammatory enzymes, such as phospholipase A2 (PLA2), cyclooxygenase 2 (COX-2) and nitric oxide synthase-2 (NOS-2). The mechanism of action of CS explains its beneficial effect on the cartilage, synovial membrane and subchondral bone. In vivo, hpCS given p.o*.* prevented hepatic NFB nuclear translocation and this suggested that systemic hpCS could elicit an anti-inflammatory effect in many tissues besides the articulation. On this basis, Ronca et al. [24] and Cohen et al. [25] reported that hpCS could be useful also in other inflammatory diseases, like psoriasis and atherosclerosis. Jomphe et al. [26] observed that the beneficial effects of hpCS in patients with OA result partially from its antinflammatory and immunomodulatory actions, as the reduction of NFB nuclear translocation, the decrease in the synthesis of pro-inflammatory cytokines IL-1 and TNF and in the activity of NOS-2 and COX-2. Other actions of hpCS contribute to its activity, such as the increase in the synthesis of articular cartilage PG, the reduction in the apoptosis of chondrocytes and the reduction of the synthesis and/or activity of MMPs [27–32].

### Clinical evidence on highly purified chondroitin sulfate

Table 2 summarizes the clinical evidence on hpCS in OA, preclinical studies and meta-analyses discussed are not included.

**Table 2:** Clinical studies on hpCS in Hip, Knee and Hand OA, on formulation 1200 mg/die and on pharmacoeconomic impact

Condition/topic First author [Ref.] Design	Patients	Treatment/dose Control/dose Follow-up	Summary of results, primary efficacy parameters
*Hip OA*			
Conrozier 1992 [33] RCT	56	hpCS 3 × 400 mg/day PLB 6 months	Pain (VAS); LI; analgesic consumption; patient’s assessment, hpCS better than PLB
*Knee OA*			
Uebelhart 1998 [34] RCT pilot	42	hpCS 800 mg/day PLB 1 year	Pain and overall mobility (VAS) at 3 (*p* < 0.05) and 12 (*p* < 0.01) months hpCS better than PLB
Uebelhart 2004 [35] RCT	120	hpCS 800 mg/day PLB 2 × 3 months during 1 year	hpCS: more LI decrease (36% hpCS vs 23% PLB; * p* = 0.001); less JSW reduction (0.44 vs 0.46 mm * p* < 0.05) than PLB
Michel 2005 [36] RCT	300	hpCS 800 mg/day PLB 2 years	JWS at 2 years: hpCS no change; PLB − 0.14 mm, (*p* < 0.001 vs. baseline)
Radrigàn 2007 [37] Open, non-controlled	61	hpCS 800 mg/day for 3 months follow-up: 6 months	Improvement of 44.4% in the LI (*p* < 0.0001) and of 56.8% (right) and 61.7% (left) in the knee pain measured by VAS
Kahan 2009 [38] RCT	622	hpCS 800 mg/day PLB 2 years	Reduction in JSW loss with hpCS (*p* < 0.0001 vs. PLB); less patients with radiographic progression in hpCS (*p* < 0.0005)
Möller 2010 [39] RCT	129	hpCS 800 mg/day PLB 3 months	hpCS better than PLB in pain at VAS (*p* < 0.01), LI (*p* < 0.05) reduction of use of analgesics (*p* < 0.05)
Wildi 2011 [40] RCT	69	hpCS 800 mg/day PLB 6 months double- blind hpCS 800 mg/ day 6 months open-label	Less cartilage volume loss in hpCS than in PLB group (*p* = 0.03). Lower subchondral BML scores in hpCS group at 12 months (lateral compartment * p* = 0.035; lateral condyle * p* = 0.044)
Montfort 2012 [41] RCT	45	hpCS 800 mg/day paracetamol 4000 mg/day 6 months	hpCS significantly reduced synovitis compared to paracetamol (*p* < 0.01) hpCS effectively reduced functional incapacity (*p* < 0.01)
Reginster 2017 [42] RCT	604	hpCS 800 mg/day celecoxib 200 mg/day PLB 6 months	At day 182 pain (VAS) reduced (*p* = 0.001 hpCS; * p* = 0.009 celecoxib) and LI reduced (*p* = 0.023 hpCS; * p* = 0.015 celecoxib) vs. PLB
*Hand OA*			
Wang 1992 [43] RCT	34	hpCS 3 × 400/day PLB 18 months	hpCS reduced pain (VAS) and improved hand function
Verbruggen 1998 [44] RCT	119	hpCS 3 × 400 mg/day PLB 3 years	Patients with new joints with lesions: hpCS 5.9%; PLB 22.4%
Rovetta 2002 [45] RCT	24	hpCS 800 mg/day + naproxen 500 mg/day naproxen 500 mg/day 24 months	hpCS + naproxen lower increase in number of joints with erosions (*p* < 0.05)
Rovetta 2004 [46] RCT	24	hpCS 800 mg/day + naproxen 500 mg/day naproxen 500 mg/day 24 months	hpCS + naproxen better than naproxen in Heberden (*p* < 0.001) and Dreiser (*p* < 0.001) scores, in patient’s (*p* < 0.001) and clinician’s (*p* < 0.001) judgement
Gabay 2011 [47] RCT	162	hpCS 800 mg/day PLB 6 months	Significant decrease in the patient’s global assessment of hand pain (difference VAS scores − 8.7 mm; * p* = 0.016) and significant improvement in FIHOA score (− 2.14; * p* = 0.008) in hpCS group vs placebo
*Condrosulf 1200 mg* *Knee OA*			
Morreale 1996 [48] RCT	146	hpCS 3 × 400 mg/day diclofenac 3 × 50 mg/day; 3 months + 3 months follow-up	LI hpCS − 64.4%; diclofenac vs − 29.7% vs baseline; paracetamol consumption hpCs − 88%; diclofenac − 37.8% (*p* < 0.01)
Bourgeois 1998 [49] RCT	127	hpCS gel 1 × 1200 mg/day hpCS 3 × 400 mg/day PLB 3 months	hpCS 1 × 1200 and 3 × 400 lower than PLB in LI (*p* < 0.0001 at day 91) and pain (VAS) (hpCS 1 × 1200 * p* < 0.01 from day 14; hpCS 3 × 400 * p* < 0.005 from day 42)
Pavelka 1998 [50] RCT	140	hpCS 200 mg/die 2 × 400 mg/die 3 × 400 mg/die PLB 3 months	hpCS 2 × 400 and 3 × 400 mg/die more effective than 200 mg/die and PLB on LI (*p* < 0.01); pain at VAS (*p* < 0.01). No difference between 2 × 400 and 3 × 400 mg/day
Clegg 2006 [51] RCT	1583	hpCS 3 × 400 mg/day GlcN 3 × 500 mg/day GlcN + hpCS celecoxib 200 mg/day PLB 2 years	Response rate, percent difference from PLB GlcN + 3.9% (*p* = 0.30), hpCS: + 5.3% (*p* = 0.17), GlcN + hpCS: % 6.5% (*p* = 0.09) celecoxib: + 10.0% (*p* = 0.008)
Zegels 2013 [52] RCT	353	hpCS 1 × 1200 mg/day 3 × 400 mg/day PLB	hpCS 1200 mg or hpCS 3 × 400 mg/day significantly improved compared to PLB in terms of LI (*p* < 0.001) and VAS for spontaneous pain (*p* < 0.01)
Pelletier 2016 [53] RCT	114	hpCS 3 × 400 mg/day celecoxib 200 mg/day 2 years	hpCS showed less cartilage loss than celecoxib in medial compartment (*p* = 0.018) and medial condyle (*p* = 0.008)
IBSA 2019 RCT	246	hpCS 1 × 1200 mg/day hpCS 3 × 400 mg/day 91 days	hpCS 1200 mg once daily not inferior to hpCS 3 × 400 mg/day in LI (− 2.9 ± 0.3; − 2.6 ± 0.3, respectively; * p* < 0.0001). No significant difference regarding pain, NSAIDs consumption
IBSA 2019 RCT	94	hpCS 1 × 1200 mg/day hpCS 3 × 400 mg/day PLB 91 days	Mean (± SD) decrease of LI from baseline to day 91: − 4.3 (3.3) in the hpCS 1200 mg group, − 4.1 (2.9) in the hpCS 400 mg group and − 1.0 (2.0) in the PLB group
*Pharmacoeconomy*			
Bruyère 2009 [58] Knee OA RCT	622	hpCS 800 mg/die PLB 2 years	Health Utility Index better for hpCS than PLB at 6 months (*p* < 0.03)
Lagnaoui 2006 [59] OA Prospective observational	844	hpCS 800–1200 mg/day Long-term (≥ 3 months) Recent (< 3 months) users	Lower consumption of NSAIDs (*p* < 0.05) and analgesics (*p* < 0.01) by long-term users
Rubio-Terres 2010 [4] OA Observational retrospective	530	CS NSAIDs CS + NSAIDs 6-months	Treatment cost 6-month: CS €141; NSAIDs €182. Concomitant CS could reduce use of NSAIDs

### Hip and knee OA

**Fig. 3:**
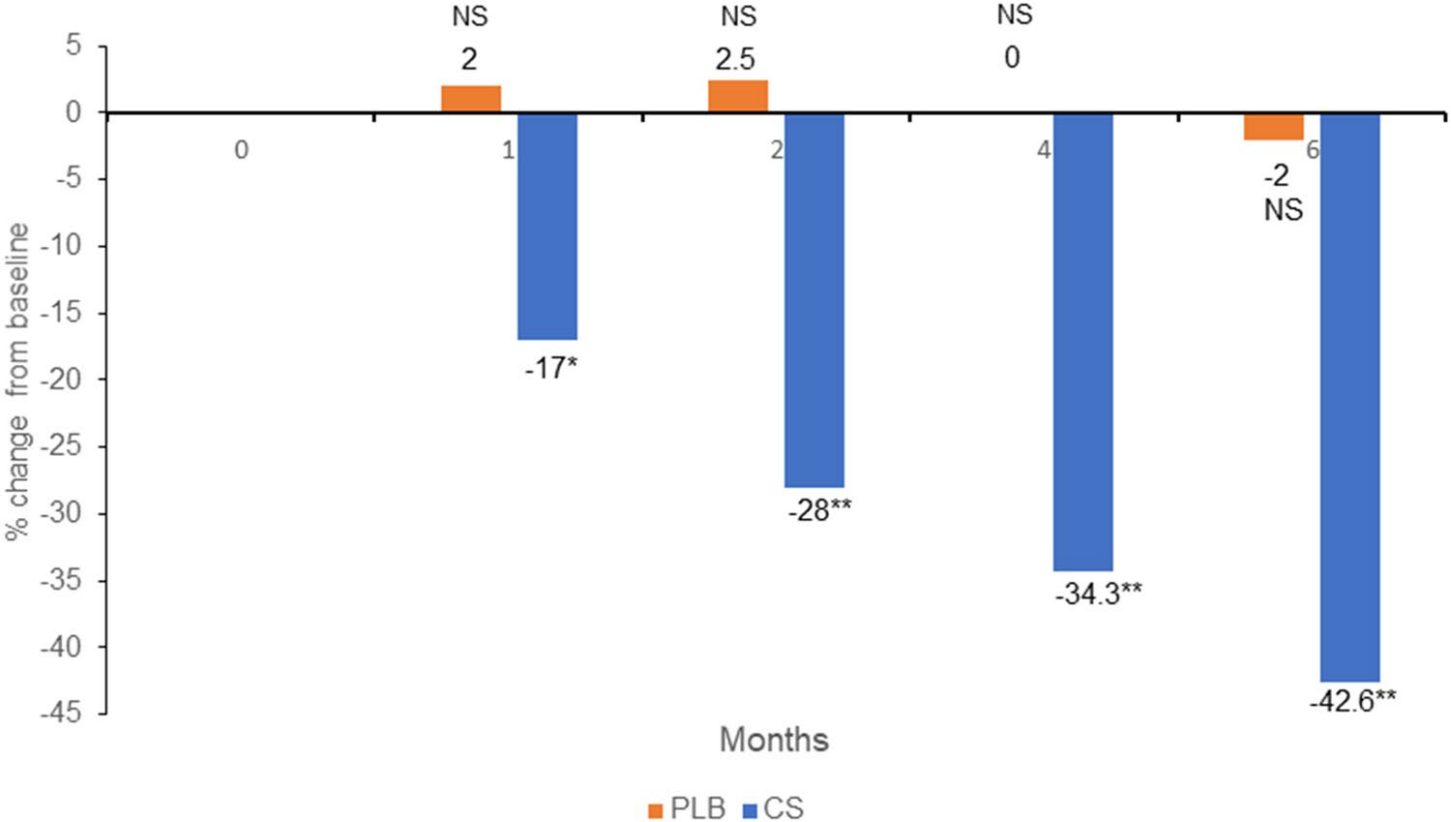
Reduction of pain in hip measured by a VAS scale; *p < 0.01, **p < 0.001, NS Not Significant; drawn based on data reported in Conrozier and Vignon [33]

Conrozier and Vignon [33] in their study on hip OA used eight parameters: (1) Huskisson's Visual Analogue Scale (VAS); (2) Lequesne's Index (LI); (3) analgesic consumption; (4) morning stiffness; (5) walking autonomy; (6) awakenings during the night, (7) intramalleolar distance, (8) patient evaluation. Parameters 1, 2, 3 and 8 were considered as essential. The hpCS group showed a statistically significant improvement compared to the placebo (PLB) in all essential parameters. Figure 3 shows the evolution of pain measured by the VAS.

As for knee OA, Uebelhart et al. [34, 35] conducted a pilot study and a RCT. In the pilot study, hpCS was associated with a stabilization of the medial femorotibial joint space width (JSW). The study evaluated also biomarkers of bone formation (osteocalcin), aggrecan (serum antigentic keratan sulfate) and connective tissue (urinary pridinolyne) degradation. The parameters were stabilized in hpCS patients, whilst they remained abnormal in the PLB group. In the subsequent RCT, LI decreased significantly by 36% in the hpCS group after 1 year compared to 23% in the PLB group (*p* = 0.001). Radiological progression at month 12 showed a significant decrease of JSW in the PLB group, whilst there were no changes in the hpCS group (*p* < 0.05). The authors concluded that, in addition to the actions on signs and symptoms, the effect of hpCS on the JSW narrowing provided further evidence of the structure-modifying properties of hpCS in knee OA. In his study, Michel et al. [36] found that 150 patients receiving PLB showed a progressive JSW narrowing, with a mean JSW loss of 0.14 ± 0.61 mm after 2 years (*p* = 0.001). The 150 patients treated with hpCS did not show any change in mean JSW (0.00 ± 0.53 mm; *p* NS); the minimum JSW narrowing showed a similar trend. The differences between groups were significant for mean (0.14 ± 0.57 mm; *p* = 0.04) and minimum JSW (0.12 ± 0.52 mm; *p* = 0.05). The study demonstrated that hpCS may slow the structural progression of knee OA. In a prospective, open study, Radrigàn et al. [37] observed at day 90 an improvement of 44.4% in the LI (*p* < 0.0001) and of 56.8% (right) and 61.7% (left) in the knee pain measured by VAS. At day 180 (90 days after the last administration of hpCS) the parameters were still significantly better than the basal levels. The residual effect was more marked in patients < 65 years and in those with less basal radiological damage. Kahan et al. [38] studied the effects of hpCS on the progression of knee OA. The hpCS group had a reduced loss of minimum JSW compared with the PLB group (− 0.07 ± 0.03 mm vs − 0.31 ± 0.04 mm *p* < 0.0001). The percentage of patients with radiographic progression > 0.25 mm was lower in the hpCS than in PLB group [28% vs 41% (*p* < 0.0005); with a relative risk reduction of 33% (95% CI 16–46%)]. The number of patients needed to treat was eight (95% CI 5–17). Pain improved faster in the hpCS than in the PLB group (*p* < 0.01).

**Fig. 4:**
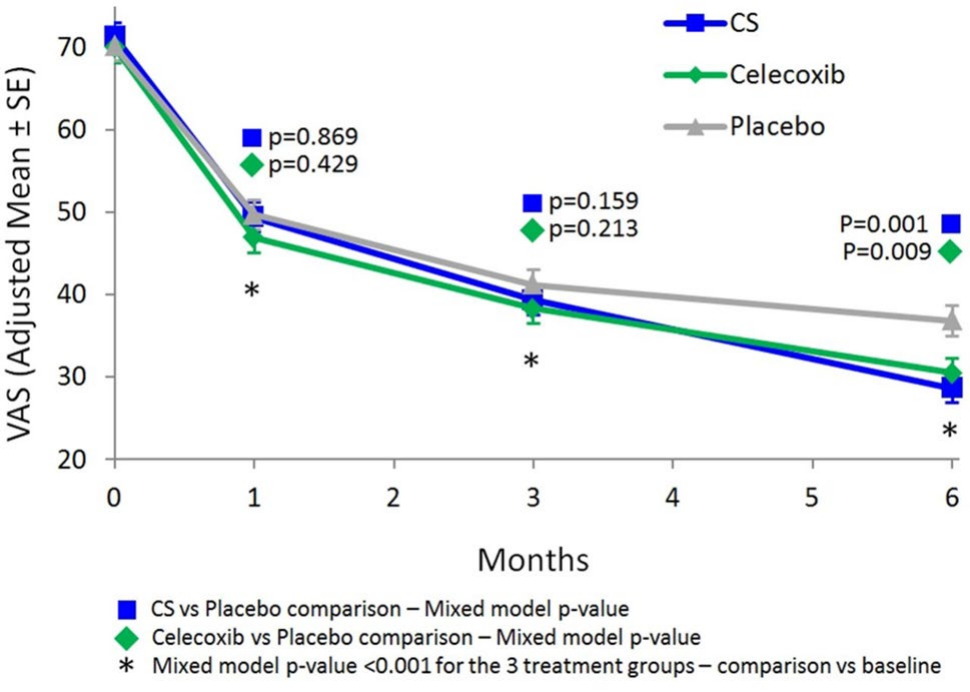
Reduction of pain in knee measured by a VAS scale. Reproduced from Reginster et al. [42] with permission from Elsevier

Möller et al. [39] found that after 3 months hpCS resulted more effective than PLB, relieving pain at VAS (hpCS − 26.9 ± 24.8; PLB − 14.23 ± 20.8 mm, *p* < 0.01), decreasing the LI (hpCS − 4.8 ± 3.4; PLB − 3.3 ± 3.5, *p* < 0.05) and reducing the use of paracetamol as rescue medication (hpCS 43%; PLB 64%, *p* < 0.05). hpCS improved also signs of plantar psoriasis more than PLB (hpCS 87%; PLB 27%, *p* < 0.05). In the RCT by Wildi et al. [40], hpCS group showed significantly less cartilage volume loss than the PLB group as early as 6 months for the global knee (*p* = 0.030), the lateral compartment (*p* = 0.015) and tibial plateaus (*p* = 0.002). The difference remained significant at 12 months. Significantly lower subchondral bone marrow lesions scores were found for the hpCS group at 12 months in the lateral compartment (*p* = 0.035) and the lateral femoral condyle (*p* = 0.044). Montfort [41] focused his study on synovitis. HpCS but not paracetamol reduced synovitis and symptoms in OA patients. The study showed also a decrease in synovial and plasma levels of inflammatory chemokines in hpCS group. In his RCT, Reginster et al. [42] observed that hpCS and celecoxib showed a significant reduction in pain and LI compared with PLB. In the intention-to-treat (ITT) population, pain reduction at VAS at day 182 was significantly greater in the hpCS group (− 42.6 mm) and in the celecoxib group (− 39.5 mm) than in PLB group (− 33.3 mm, *p* = 0.001 hpCS; *p* = 0.009 celecoxib), without difference between hpCS and celecoxib (Fig. 4). The improvement of LI was greater in the hpCS (− 4.7) and celecoxib (− 4.6) than in the PLB group (− 3.7) (*p* = 0.023 for HPCS; *p* = 0.015 for celecoxib). No difference was observed between hpCS and celecoxib. The secondary endpoints: Minimal-Clinically Important Improvement (MCII) and Patient-Acceptable Symptoms State (PASS), showed significant improvement in the hpCS and celecoxib groups compared to PLB.

### Hand OA

Wang et al. [43] on 34 patients found that the treatment with hpCS reduced pain and improved function of the hand without impact on radiological signs. In the RCT by Verbruggen et al. [44], hpCS group reported a significant decrease in the number of patients with new erosive OA lesions in the finger joints. In two RCTs, Rovetta et al. [45, 46] observed a significant difference in favour of the hpCS + naproxen group compared to naproxen only in number of joints with erosions (*p* < 0.05), a superiority of hpCS in Heberden (*p* < 0.001) and Dreiser (*p* < 0.001) scores and in patient’s (*p* < 0.001) and clinician’s (*p* < 0.001) judgement. Gabay et al. [47] found that, compared with PLB, patients treated with hpCS showed a significant decrease in the patient’s global assessment of pain (difference VAS scores − 8.7 mm; *p* = 0.016). The hpCS group showed also a significant improvement in Functional Index for Hand OsteoArthritis (FIHOA) score (− 2.14; *p* = 0.008), in morning stiffness and in the investigator’s global impression of efficacy.

### Condrosulf 1200 mg/day

The recommended dose of hpCS is 800 mg/day; yet in the most severe cases an initial dose of 1200 mg/day is advisable for the first weeks of treatment, which is then followed by a reduction of the dose to 800 mg. Morreale et al. [48], comparing hpCS with diclofenac observed that diclofenac had a faster effect, but the treatment with hpCS 3 × 400 mg/day was associated with longer-lasting symptomatic efficacy, with a lower LI from day 60 to day 180. Bourgeois et al. [49] found that the physician’s and patient’s overall efficacy assessments were superior for hpCS than PLB (*p* < 0.001). The efficacy of hpCS 1200 oral gel mg once daily did not differ from that of hpCS 3 × 400 mg/day. Pavelka et al. [50] studied three dosages of hpCS and found that hpCS at 800 and 1200 mg/day was more effective than PLB without difference between the two doses, whilst hpCS 200 mg/day was not different form PLB. Clegg et al. [51] reported that hpCS and GlcN, alone or in combination, had a modest effect on pain reduction compared to PLB, which was associated with a high rate of response. Despite the marked PLB effect, the response rate to hpCS in monotherapy was 5.3% points higher than PLB. In the RCT by Zegels et al. [52], after 3 months, no significant difference was observed between hpCS 1200 mg oral gel once daily and hpCS 3 × 400 mg/day. Patients treated with hpCS 1 × 1200 mg/day or 3 × 400 mg/day significantly improved compared to PLB in terms of LI (*p* < 0.001) and VAS for spontaneous pain (*p* < 0.01). Pelletier et al. [53] demonstrated that hpCS was superior than celecoxib in reduction of cartilage volume loss measured by magnetic resonance. HpCS and celecoxib were similarly effective in improving joint effusion and/or swelling, WOMAC total score, WOMAC pain score, pain assessed by VAS and quality of life (Short Form-36). Two phase III, multicenter RCTs (IBSA, data on file) evaluated the efficacy and safety of hpCS 1200 mg oral gel once daily vs hpCS 3 × 400 mg/day. The first study showed that hpCS 1200 mg once daily was not inferior to hpCS 3 × 400 mg/day in LI (− 2.9 ± 0.3; − 2.6 ± 0.3, respectively; *p* < 0.0001). No significant difference between groups was observed regarding pain, NSAIDs consumption or patient and investigator global assessment of efficacy. The second study confirmed these evidences. The improvement in LI and the decrease in pain were significantly greater in both hpCS groups compared to PLB, without differences between the two hpCS formulations.

### Safety

The overall analysis of all the studies included in this review, provided evidence that hpCS formulations have a good safety profile. The incidence and severity of hpCS-related adverse events (AEs) are low and similar to those of the placebo also at the dosage of 1200 mg/day. Gastrointestinal disorders were the most common AEs and were reported more in patients of the PLB group than in those of the two hpCS groups with statistically significant differences. Both patients and investigators expressed an excellent/good opinion of safety in the vast majority of cases. These data are consistent with the worldwide post-marketing data from Europe, Middle East, North and South America since 1982 (IBSA data on file).

### Chondroitin sulfate versus food supplements

Food supplements (FS) do not undergo the strict quality controls of pharmaceuticals, because of loose regulatory constraints; this raises concerns about their safety and clinical efficacy. As stated in the position paper endorsed by ESCEO [54], only pharmaceutical CS is shown to deliver consistently high CS bioavailability and plasma concentration in humans, which corresponds to demonstrated clinical efficacy. Volpi and Maccari [55] assessed the amount, quality and origin of CS from ten Czech Republic FS preparations. Only four of the ten preparations met the label specifications. Other four preparations contained between 0 and 1% CS in comparison with the contents declared on the label (47%, 17%, 12%, 6%). Two preparations had 30–45% of the declared content of CS, and one contained approximately 2% HA. The CS contained in eight FS was bovine or porcine, in one preparation CS derived from cartilaginous fish and in one case CS levels were too low for any determination.

Restaino et al. [56] examined 25 FS preparations from eight European countries by multiple analytical methods (high performance chromatography, nuclear magnetic resonance, capillary electrophoresis) and biological assays (action on chondrocyte culture). The FS were then compared to two pharmaceutical CS products. Compared to the pharmaceutical-grade products, FS contained low-quality CS, in some cases of multiple animal origins and of dis-homogeneous molecular weights. The FS resulted to be highly contaminated by keratan sulfate; the presence of high insoluble solids and solvent residues was suggestive of poorly controlled manufacturing procedures or low-quality raw materials used in the FS preparations. Stellavato et al. [57] assessed the purity, the titer, and the origin of ten different FSs containing CS and then compared their biological activity with two pharmaceutical CS products. The pharmaceutical-grade products demonstrated an effective modulation of biomarkers counteracting the inflammation status and improving viability and the physiological condition of OA human primary chondrocytes and synoviocytes. In contrast, most FSs were cytotoxic at the tested concentrations, and only three out of ten FSs had an in vitro behaviour similar to that of the pharmaceutical-grade products.

The results of the above mentioned researches demonstrate the need for stricter rules to control the quality of FSs to obtain safe and effective products.

### Pharmacoeconomy

HpCS was comprehensively studied from the pharmacoeconomic point of view. Bruyère et al. [58] in a 2-year RCT, evaluated the impact of hpCS on health-related quality of life using utility values in patients with knee OA. The Health Utility Index (HUI) score changes from baseline to 6 months were 0.02 ± 0.02 and 0.05 ± 0.01 for the PLB and hpCS groups, respectively (*p* = 0.03). After 24 months, the HUI score increased by 0.04 ± 0.02 in the PLB group and 0.05 ± 0.02 in the hpCS group (*p* = 0.37). Considering the price of hpCS in Europe, the cost effectiveness ratio assessment always resulted in a cost below € 30,000 per quality-adjusted-life-year (QALY) gained, after 6, 12 and 24 months of treatment. Lagnaoui et al. [59] studied the impact of the use of hpCS 400 mg on the consumption of analgesics and NSAIDs. Patients with OA (844) OA were divided in “recent hpCS users” (≤ 30 days of continuous use; 222 pat.) and “long-term hpCS users” (> 30 days of continuous use; 622 pat). Ninety-eight (11.6%) patients did not use any analgesic or NSAIDs; 746 (88.4%) reported the use of at least one of these drugs. Compared to recent users, long-term users of hpCS400 had a significantly lower current (44.4 *vs.* 52.5%, *p* < 0.05) and long-term use of NSAIDs (11.8% versus 18.5%, *p* < 0.05) and of analgesics (70.3 versus 79.3%, *p* < 0.01). The results suggested that the use of hpCS could reduce the consumption of NSAIDs. The retrospective study conducted by Rubio-Terrés [4] (data from the VECTRA study) evaluated the economic impact of the treatment with hpCS or NSAIDs. The use of hpCS in 530 patients with OA for more than 6 months reduced the use of NSAIDs. The overall 6-month cost per patient was 141€ for CS and 182€ for NSAIDs. The authors estimated that in 3 years a gradual shift to hpCS of 5%, 10%, and 15% of patients currently treated with NSAIDs could generate 38,700,000€ savings for the Spanish National Health System. In addition, the authors calculated that, for every 10,000 patients switching from NSAIDs to hpCS 2666 cases of gastrointestinal AEs (including 90 SAE) could be avoided.

## Discussion

This paper provides an updated and comprehensive overview of efficacy, safety, quality and health economics impact of the treatment with hpCS (Condrosulf^®^, IBSA). In addition to this, unpublished efficacy data on the new formulation oral gel 1200 mg once daily are presented. The main limit of the paper lies in the different design, size and assessment measures (e.g. radiological techniques) of individual trials; especially the methods of older studies were hampered by the period of their publication. The review of the scientific literature has raised some points that in our opinion deserve further investigation: characteristics of the structural progression of the disease and the most appropriate methods to assess and quantify it; the role of the treatment with hpCS combined with other SYSADOAs (e.g. intra-articular hyaluronic acid) or NSAIDs; biomarkers or predictors of response to treatment with hpCS.

## Conclusion

Highly purified CS, at 400 mg, 800 mg and 1200 mg was extensively studied. It proved to be safe and effective in hip, knee and hand OA, acting on signs, symptoms and structural changes. The use of hpCS reduced the use of NSAIDs and their side effects. Furthermore, the use of hpCS was found to be cost-effective up to a period of 24 months. It is also important to point out that the quality of hpCS is the pre-requisite for safe and effective preparations.
